# Au-Graphene Hybrid Plasmonic Nanostructure Sensor Based on Intensity Shift

**DOI:** 10.3390/s17010191

**Published:** 2017-01-19

**Authors:** Raed Alharbi, Mehrdad Irannejad, Mustafa Yavuz

**Affiliations:** Waterloo Institute for Nanotechnology, University of Waterloo, Waterloo, ON N2L 3G1, Canada; r2alharb@uwaterloo.ca (R.A.); myavuz@uwaterloo.ca (M.Y.)

**Keywords:** hybrid nanostructure, graphene, LSPR sensor, intensity shift, sensitivity

## Abstract

Integrating plasmonic materials, like gold with a two-dimensional material (e.g., graphene) enhances the light-material interaction and, hence, plasmonic properties of the metallic nanostructure. A localized surface plasmon resonance sensor is an effective platform for biomarker detection. They offer a better bulk surface (local) sensitivity than a regular surface plasmon resonance (SPR) sensor; however, they suffer from a lower figure of merit compared to that one in a propagating surface plasmon resonance sensors. In this work, a decorated multilayer graphene film with an Au nanostructures was proposed as a liquid sensor. The results showed a significant improvement in the figure of merit compared with other reported localized surface plasmon resonance sensors. The maximum figure of merit and intensity sensitivity of 240 and 55 RIU^−1^ (refractive index unit) at refractive index change of 0.001 were achieved which indicate the capability of the proposed sensor to detect a small change in concentration of liquids in the ng/mL level which is essential in early-stage cancer disease detection.

## 1. Introduction

Sensors based on surface plasmon resonance (SPR) phenomena have a potential application in chemical/bio sensing and gas detection [1,2]. When a nanoparticles (NP) with a subwavelength particle size exposed to an incident electromagnetic field, free electrons in the conduction band oscillate at the surface of the NP and the surrounding dialectic medium interface at a wavelength called the plasmonic resonance wavelength. This phenomenon called localized surface plasmon (LSP) [3,4]. The resonance position and the intensity of the LSP are sensitive to the refractive index changes of the surrounding medium [5], which is the basic principle of the plasmonic sensors operation. When the surrounding’s refractive index changes, the resonance peak wavelength shifts from its initial position. The intensity of the resonance mode may also shift as reported elsewhere [6]. The sensitivity of a localized surface plasmon sensor can be obtained by calculating the ratio of the resonance wavelength shift (or resonance intensity shift) at a given ambient refractive index change (Δn), called bulk sensitivity, S_λ_, (intensity sensitivity, S_I_) [7,8]. According to Mie theory [9,10], geometrical properties of a NP such as shape, size and materials would affect the resonance properties of the LSPR. Kumar et al. [11] reported that increasing in the Ag NPs size enhances the intensity of the plasmon resonance and, hence, improves the sensitivity of the plasmonic-based sensor. In addition to the geometrical effect, chemical stability of the NPs is also importance to achieve good performance of the plasmonic device, as reported in [12,13]. Furthermore, using nanostructures in arrays could enhance the interaction between the incident electromagnetic field and free electrons, which leads to improvements in the plasmonic properties of the desired nanostructure [14,15,16]. In addition to the simplicity and low fabrication cost LSPR sensors offer larger sensitivity to the local refractive index change compare to the propagated surface plasmon resonance (PSPR) ones [8,17]. It is known that the LSPR sensors suffer from low bulk sensitivity and large full width at half maximum (FWHM) of the resonance mode, which is attributable to the radiative damping mode of the NPs. The radiative damping mode of NPs results in a reduction of the figure of merit (i.e., FOM = Sensitivity/FWHM [8,18,19]) of a sensor. Different approaches were employed to improve the performance of the LSPR sensor by enhancing the bulk sensitivity and reducing the FWHM of a resonance modes [15,16,20]. 

Integrating the metallic nanostructures with two-dimensional (2D) materials (graphene-like materials), such as graphene, attracted the plasmonic research community interest for their capability to enhance the resonance properties of the hybrid structure [21,22,23]. Integrating graphene layers with conventional plasmonic nanostructures is a promising approach to design a nanostructure with a larger FOM. By employing graphene as a spacer between the Au film and Au nanoparticles, FOM as large as 2.8 can be achieved relative to the structure without graphene spacer (FOM = 2.1) [22]. Encapsulating Au NPs with Ag NPs, and vice versa [24], can also results in a FOM of 2.7. In our previous work, we report the FOM as large as 102 in the 2D periodic nanostructure of Au-graphene core shell nanospheres [6] which is close to the theoretical limit using a mushroom Au NP periodic array [25]. However, more improvement in the FOM is required in LSPR sensors to overcome low performance compared to the propagating SPR sensor. 

**Figure 1:**
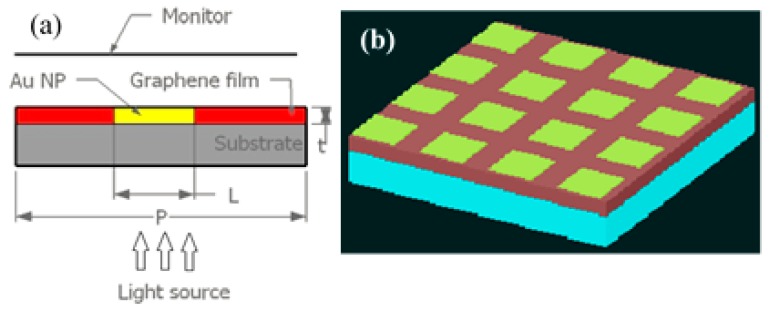
(**a**) 2D schematic diagram of proposed sensor used in this study. L is the side length of cubic and prism NPs, and the diameter of the cylindrical NPs, t is the NPs thickness, and P is the separation distance between two consecutive NPs; and (**b**) the perspective view of the Au-graphene hybrid sensor.

In this work, a 2D periodic structure of imbedded Au NPs with different shapes in a multilayer graphene film was studied as a plasmonic sensor. A two-dimensional array of Au NPs with different shapes (i.e., cubic, cylindrical, and prism) were fabricated on a quartz substrate and the gap between the NPs was filled with multilayer graphene film, as schematically shown in Figure 1. A series of nanostructure arrays were systematically studied to obtain a sensor device with larger FOM and bulk sensitivity simultaneously. According to our previous study [6] the center-to-center distance between two consecutive particles was chosen as 300 nm and reduced by increasing the Au NP dimension from 50 nm to 300 nm. The size to separation distance ratio (L/P) was varied from 0.17 to 1 and the thickness of the nanostructure was fixed at 20 nm. The L and P are the NPs lateral size and structural periodicity.

The extinction spectrum was calculated at the near-infrared (NIR) region (λ = 1.5 to 2 μm) which is a desired region for detection of biomarkers (e.g., breast cancer biomarkers) using blood serum [26,27]. The refractive index of the sensing medium was varied from 1.333 (water) to 1.341 with a step of 0.001 to measure the ability of the proposed nanostructure sensor to detect small variations in concentration at the ng/mL level [28]. The proposed sensor can be fabricated by growing Au NP arrays using electron beam lithography [29] or using a focused ion beam (FIB) [30] to produce nanohole arrays in graphene films and then filling the holes with the NPs using the electron beam deposition method.

## 2. Numerical Methodology 

The finite difference time domain (FDTD) method is a powerful method to solve the Maxwell’s equations in a nanostructure with arbitrary and symmetrical structures by using the YEE-algorithm [31]. The FDTD method is a more reliable method than others, such as multiple-multiple method or Green’s dynamics method in solving Maxwell’s equations of complex geometry and dispersive media, such as gold and silver [7,32,33]. In this study, the FDTD method was employed to study the extinction properties and sensitivity of a series of nanostructures with different geometrical parameters fabricated on a glassy substrate. The refractive index of the substrate was considered as 1.45 and the refractive index of the superstrate (i.e., target materials for sensing application) was varied from 1.333 to 1.341 with a 0.001 step to cover the refractive index change range of the solution with deoxygenated hemoglobin (HB) in phosphate-buffered saline solution up to 60% concentration (g/L) as reported by O. Zhernovaya et al. [34]. Each layer was specified by electrical permittivity, ε(ω). The substrate permittivity was considered as 2.1025 (*n* = 1.45). The Lorentz-Drudee model were employed to describe the gold nanoparticles and graphene permittivity over the studied wavelengths [35,36]:

(1) $$\varepsilon \left(\omega \right)=\varepsilon_{\infty }+\overset{M}{\underset{0}{\sum }}\frac{f_{m}\omega_{p}^{2}}{\omega_{m}-\omega^{2}+i{\omega \Gamma }_{m}}$$

where ε_∞_ is the permittivity in the infinity frequency, ω and ω_p_ (1.37188 × 10^16^ rad) are the incident and gold plasma frequencies, respectively. The ω_m_ is the mth resonance frequency, and Γ_m_ is the *m*th damping frequency which is obtained by fitting the empirical data for real and imaginary parts of gold.

The Falkovsky model was employed to calculate the graphene permittivity tensor in XY plane which is given by [37]:

(2) $$\varepsilon_{xx}=\varepsilon_{yy}=\varepsilon_{r}-\frac{e^{2}\mu_{0}}{\pi \hbar^{2}}\frac{\omega }{\omega^{2}+{\left(\frac{ev_{f}^{2}}{\mu_{0}\mu }\right)}^{2}}\frac{1}{{\omega \varepsilon }_{0}\Delta }+i\frac{e^{2}\mu_{0}}{\pi \hbar^{2}}\frac{1}{\omega^{2}+{\left(\frac{ev_{f}^{2}}{\mu_{0}\mu }\right)}^{2}}\frac{ev_{f}^{2}}{{\omega \mu \mu }_{0}\varepsilon_{0}\Delta }$$

where $\varepsilon_{r}$ is the background relative permittivity, *e* is the electron charge(C), $\mu_{0}$ is the chemical potential (joule) (Fermi energy), ℏ is the reduced plank constant, ω is the angular frequency of incident photon, $v_{f}$ is the Fermi velocity (m/s), μ is the carrier mobility (m^2^/VS), $\varepsilon_{0}$ is the vacuum permittivity (F/M), and Δ is the graphene thickness (m).

The commercial FDTD packages typically use different models, such as Drude model, Lorantz model, or Debye model to calculate the electrical permittivity of the dispersive materials like noble metals. However, while these models offer good insight into the behavior of materials permittivity, they fail to accurately capture the dispersive properties of real materials, which are impacted by impurities and defects. These limitations can be addressed by using combination of these models (e.g., Lorentz-Drude) or multipole models, such as the multipole Lorentz model; however, some materials cannot be described by these models. In this work, the multi coefficient method (MCM) from Lumerical [38] was employed to obtain high accurate electrical permittivity of the dispersive materials used in this study.

The numerical analysis was carried out using the FDTD package from Lumerical Inc. The Au-G hybrid nanostructure, plane wave source, and transmission/reflection monitors were co-planar with the boundary conditions that made them infinite in x and y directions as schematically shown in Figure 1a. A non-polarized plane wave source with emitting wavelengths in the range of 0.4 μm to 2 μm with electric field amplitude of 1 V/m was propagated along z-axis as incident light source at normal incidence. In order to reduce the calculation time, the periodic boundary conditions in the *x*- and *y*-directions were replaced by the asymmetric and symmetric boundary conditions in the *x*- and *y*-directions, respectively [39]. The perfect matching layer (PML) boundary condition was chosen in the *z*-axis to avoid electromagnetic reflection to the structure and transmission monitor. The mesh cell size (point to point distance) was 5 nm in the *x* and *y* direction and 2 nm in the *z*-direction, and the calculation time was set as 3000 fs. The transmission spectra were calculated using an *x*-*y* monitor at 150 nm away from the Au-G/superstrate (liquid) interface. The plane wave source was placed 150 nm below the structure. Air (*n* = 1) was chosen as the background and water (*n* = 1.333) was used as reference medium in the sensitivity and FOM measurements. The reflection spectra were calculated using an *x*-*y* monitor at 150 nm below the source position.

## 3. Results and Discussion

### 3.1. Contribution of the Hybrid Au-Graphene (Au-G) Nanostructure

**Figure 2:**
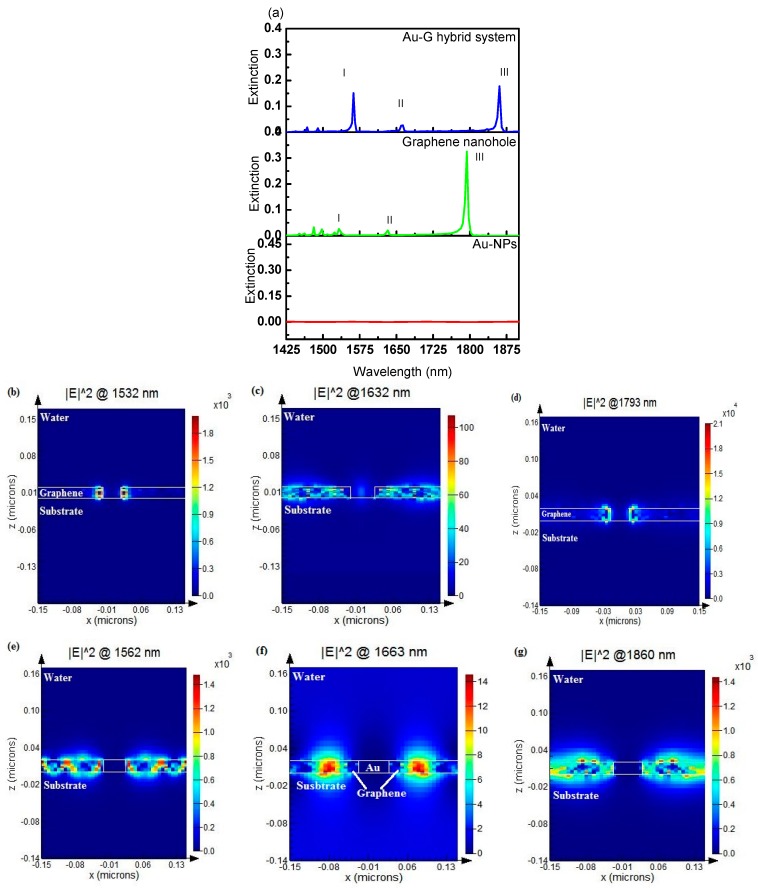
Top row: (**a**) extinction spectrum for three different nanostructures; Au NP square array, nanohole array perforated in 20 nm thick graphene film, and Au NPs/G hybrid structure. Middle row: electric field profiles at the resonance wavelengths of (**b**) 1532 nm (mode I), (**c**) 1632 nm (mode II), and (**d**) 1793 nm (mode III) of the graphene nanohole structure. Bottom row: Au-graphene hybrid structure at resonance wavelengths of (**e**) 1562 nm (mode I); (**f**) 1663 nm (mode II), and (**g**) 1860 nm (mode III), respectively.

The Au-G hybrid sensor performance were compared with a 2D array of Au NPs placed on a silica substrate, perforated square nanohole arrays in a multilayer graphene film on the silica substrate and Au-G hybrid structure fabricated on a silica substrate. In the first structure, a series of nanohole square arrays with side length of L = 50 nm, thickness of 20 nm, and periodicity of 300 nm were perforated in the multilayer graphene sheets on the silica substrate. In the second case, Au NP arrays with dimensions equal to the size of the nanohole arrays (first structure) and the same structural parameters were studied. Since the reflection loss in the studied structures were negligible (~1%), the extinction spectrum of each structures was calculated using 1 − T instead of using 1 − (T + R). Figure 2a shows the extinction spectra of a 2D nanohole array perforated in the multilayer graphene films (green curve), cubic Au NPs array (red curve), and cubic Au NPs/graphene film hybrid structure (blue curve) over a wavelength range from 1500 nm to 2000 nm. As can be seen from this figure no resonance modes were observed in the Au cubic NPs square array, however, three different resonance modes were recorded in the nanohole array structure at wavelengths of 1532 nm, 1632 nm, and 1793 nm which labeled as resonance mode I, II, and III, respectively. The maximum extinction of 0.3 was obtained at the resonance wavelength of 1793 nm. It was found that by filling the graphene nanohole with Au NPs the resonance wavelengths were shifted by 19 nm, 24 nm, and 67 nm, respectively, for resonance modes I, II, and III as shown in Figure 2a. The recorded red shift in the resonance modes can be attributed to the increasing in the refractive index of the surrounding medium (hole + sensing medium) by adding the Au NP in the nanohole. Before adding the Au NP, the surrounding’s refractive index was 1.333 (water) and adding the Au NPs results in increases in the effective refractive index. According to Equation (3), increasing in the refractive index of the surrounding medium causes a redshift in the resonance wavelength [40]:

(3) $$\lambda ={\left[\left(\frac{m}{a_{x}}\right)+\left(\frac{n}{a_{y}}\right)\right]}^{-\frac{1}{2}}\sqrt{\frac{\varepsilon_{m}n_{a}^{2}}{\varepsilon_{m}{+n}_{a}^{2}}}$$

where *ε_m_* is the permittivity of dispersive material (graphene and Au-G hybrid), *n_a_* is the refractive index of the dielectric medium, m and n are integers resonance orders, and *a_x_ = a_y_ = P* is the structural period of the array. 

Figure 2a also shows the extinction intensity of resonance mode III was reduced (from 0.3 to 0.2) on filling the nanoholes in the graphene film with Au NPs, whilst, the extinction intensity of the resonance modes I and II were increased. The resonance modes in the perforated holes in the graphene sheets were excited due to the extraordinary optical transmission (EOT) effect [40]. By using a nanohole array two different types of resonances modes are expected; a resonance mode from the nanohole/substrate interface (bottom of the structure) and another one from the nanohole/superstrate interface (top of the structure) [41,42,43]. Therefore, it is expect that resonance mode III was belong to the mode created at the hole/superstrate interface while modes I and II were recorded due the surface plasmon resonance at the hole/substrate interface [44]. 

Figure 2b–d show the electric field profiles at the resonance wavelengths of 1532 nm, 1632 nm, and 1793 nm respectively, for a square nanohole array of a multilayer graphene film with 50 nm width and 20 nm thickness. The effects of filling the nanoholes with Au on the electric field profile at the resonance wavelengths in the Au-G hybrid structure (Figure 1b) are compared in Figure 2e–g. From these figures, it is clear that the quadrupole resonance is the main responsible for resonance mode excitation in the Au-G hybrid structure. It was found that the electric field was localized at the edges of the nanoholes in the y-direction (Figure 2b–d) while it was propagated along x-direction in the hybrid structure (Figure 2e–g). It was found that filling the nanoholes with cubic NPs resulted in a significant reduction in the maximum recorded electric filed, |E|, of the nanostructure from 12 V/m to 6.11 V/m.

### 3.2. Effects of Au NP Size, Shape, and Structural Periodicity on Extinction

**Figure 3:**
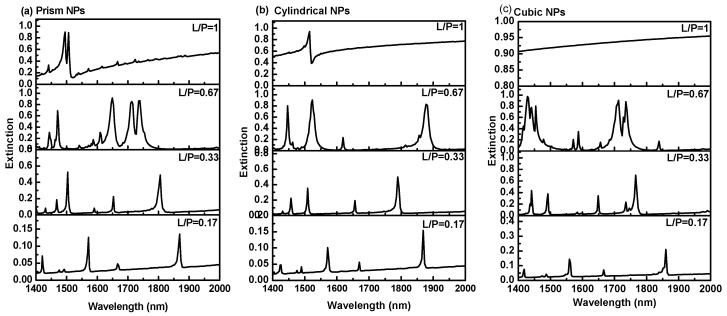
Extinction spectra of hybrid nanostructure with (**a**) prism; (**b**) cylindrical; and (**c**) cubic NPs at different L/P ratios and thickness of 20 nm.

The effects of Au NPs’ size, shape and the separation distance (P) on the extinction spectrum were systematically studied using numerical analysis. A series of Au NPs with different shapes (cylindrical, prism and cubic), different particle size, L, in the range of 50 nm to 300 nm (L/P ratio in the range of 0.17 to 1) and a fixed height of 20 nm were investigated. Figure 3 shows the extinction spectra of the resonance modes of the hybrid Au-G nanostructure with prism, cubic, and cylindrical NPs at different L/P ratios. The effects of increasing the L/P ratio and using prism Au NPs on the extinction of the Au-G hybrid nanostructure are compared in Figure 3a. As can be seen from this figure, increasing the L/P ratio from 0.17 to 1 resulted in a blueshift as large as 363 nm in the resonance wavelength of resonance mode III, which can be confirmed in Equation (3). A similar trend was also observed by using the cylindrical and cubic NPs as it is evident from Figure 3b,c, respectively. 

**Figure 4:**
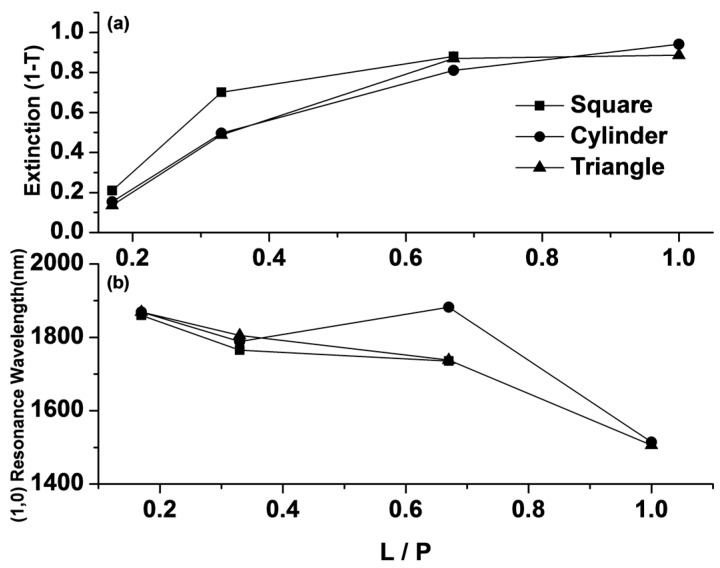
(**a**) Extinction and (**b**) (1,0) resonance wavelength as a function of the L/P ratio. L is the side length and P is the separation distance between two consecutive NPs.

It was also found that on increasing the L/P ratio, the extinction intensity of resonance mode III (the (1,0) resonance mode in Equation (3) with *m* = 1, *n* = 0) was increased in all three Au-G hybrid structures, as clearly shown in Figure 4a. It is known that graphene has a constant absorption coefficient in the visible to NIR range [45], therefore, it can be concluded that increasing in the extinction intensity on increasing the L/P ratio could attributable to the Au NP absorption coefficients where larger NPs resulted in more extinction. This could also be due to the smaller covered area by graphene film (at larger L/P ratio) and, hence, a lower amount of electron flow between two consecutive NPs. 

A maximum extinction peak intensity of 0.94 was achieved at L/P = 1 in the hybrid nanostructure with cylindrical NPs at a resonance wavelength of 1514 nm, while there are no extinction peaks in the recorded spectrum for nanostructure at L/P = 1 with cubic NPs. This is because at L/P = 1, the cubic NPs become a continuous thin film. As a result, only one resonance mode will appear at a visible range that contributes to the intrinsic absorption of gold. Figure 4b shows the mode III resonance wavelength position of all hybrid systems as a function of the L/P ratio. Figure 4b shows the (1,0) resonance wavelength position of all hybrid systems as a function of L/P ratio. It is expected that increasing the L/P ratio resulted in a blueshift in the recorded resonance wavelength in the hybrid nanostructures as show in Figure 4b and could be confirmed by Equation (3).

### 3.3. Sensitivity Measurement 

**Figure 5:**
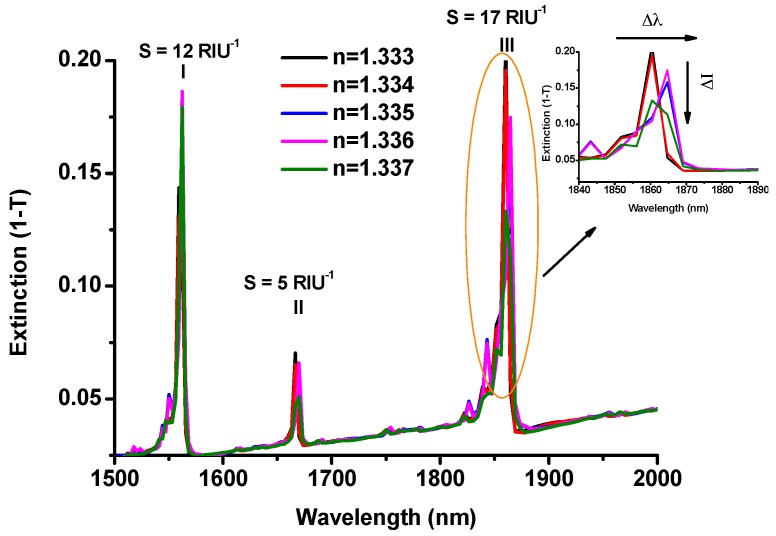
Extinction spectrum of a hybrid nanostructure with cubic NPs side at L/P = 0.17.

The plasmonic resonances wavelength and, hence, the sensitivity of a plasmonic sensor strongly depends on the refractive index variation of the surrounding environment. The optical response of an Au-G hybrid structure with cubic NPs to the refractive index variations in the range of 1.333 to 1.337 is shown in Figure 5a. As it is clear from this figure, there is not any comparable shift in the resonance wavelength on increasing the refractive index at all three resonance modes. However, the extinction intensity of the resonance modes was changed. Therefore, the intensity shift can be used to measure the sensitivity of the proposed sensor. The intensity sensitivity is calculated by dividing the change in the extinction intensity to the refractive index change (S_I_ = ΔI/Δn) [7]. As shown in Figure 5a, the intensity sensitivity at longer resonance wavelength is larger than the shorter one. The intensity sensitivity at λ = 1860 nm was measured as 17 RIU^−1^ while at shorter resonance wavelength (λ = 1562 nm), it was decreased to 12 RIU^−1^. Thus, the resonance mode at 1860 nm was chosen to study the effect of using different geometrical structures on the sensitivity and FOM of the Au-G hybrid refractive index sensor. 

The intensity sensitivity and FOM of different sensors with three different NP shapes (cubic, cylindrical, and prism) with different L/P ratios in the range 0.17 to 1 (side length, L, was varied in the range of 50 to 300 nm) and a fixed thickness of 20 nm was studied using the third resonance mode (λ = 1860). The extinction, sensitivity, and FOM of all sensors were calculated on increasing the refractive index from 1.333 to 1.341 with an increment step of 0.001. In the case of using intensity shift of the resonance peak, the FOM was calculated by dividing the sensitivity of the resonance mode of the target liquid to the resonance intensity at the reference point (FOM = S_I_/I_resonance_).

**Figure 6:**
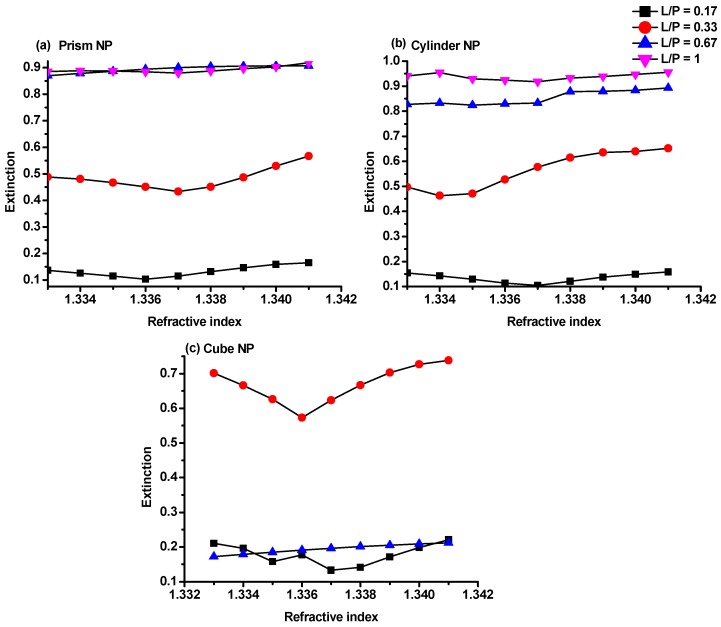
Extinction intensity of the hybrid Au-G nanostructure with (**a**) prism; (**b**) cylindrical; and (**c**) cubic NPs at different L/P ratios as a function of refractive index.

It was found that increasing the L/P ratio resulted in an increase in the extinction intensity in all different NPs shapes as shown in Figure 6 which could be attributed to the stronger absorption of the incident light by Au NPs at larger L/P [25]. As can be seen from Figure 6b, the maximum extinction intensity of 0.95 was recorded on using the cylindrical NPs with diameter of 300 nm and 20 nm height. Whereas the lowest extinction intensity was recorded in the Au-G hybrid structure using cubic NPs with side length of 300 nm as it is evident from Figure 6c. From these figures, it is also clear that there were no monotonic increases in the extinction as the L/P increase. In an Au-G hybrid structure with cubic NPs the maximum extinction was recorded as large as 0.7 at L/P = 0.33 while, by using the prism NPs, it was increased to 0.9 at L/P = 0.67 as shown in Figure 6a,c.

**Figure 7:**
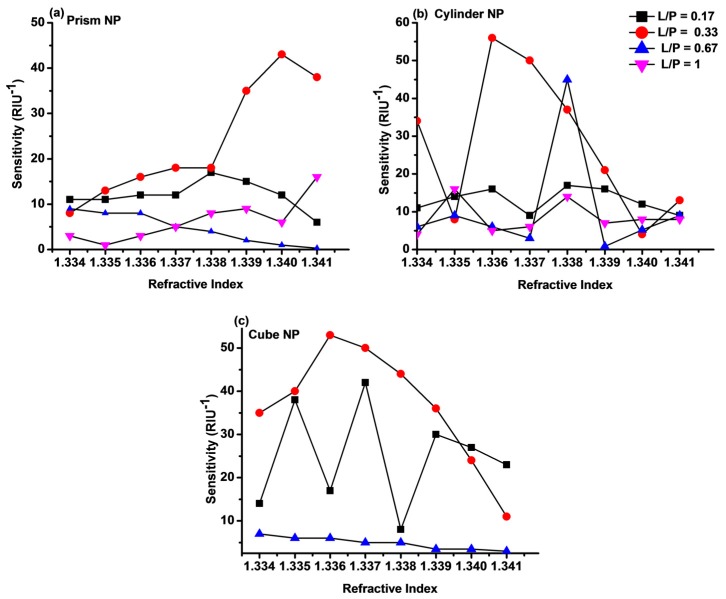
Sensitivity of the hybrid Au-G nanostructure with (**a**) prism; (**b**) cylindrical; and (**c**) cubic NPs at different L/P ratios as a function of the refractive index.

**Figure 8:**
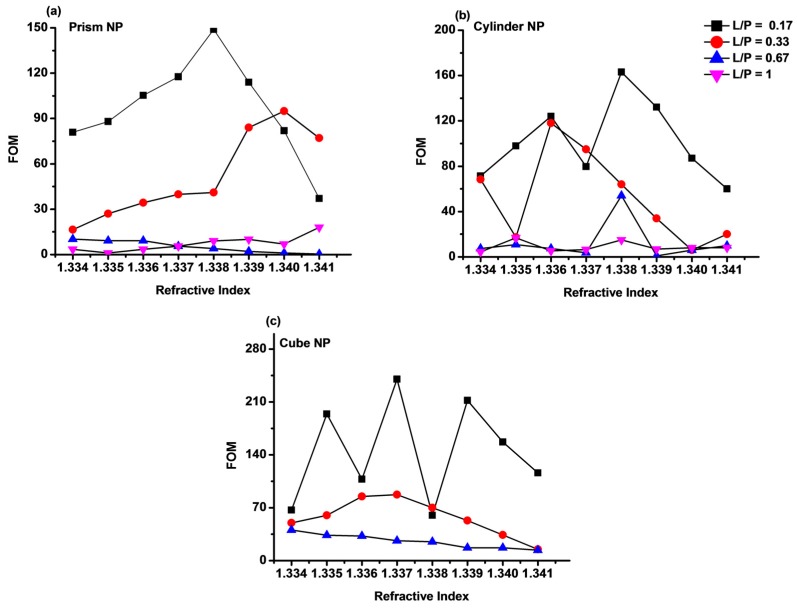
FOM of the hybrid Au-G nanostructure with (**a**) prism; (**b**) cylindrical; and (**c**) cubic NPs at different L/P ratios as a function of the refractive index.

The variation of sensitivity (S_I_) of Au-G hybrid sensor on increasing the refractive index change at different L/P ratios in all three types of Au-G hybrid sensors were compared in Figure 7. As can be seen the maximum sensitivity of 43 RIU^−1^, 56 RIU^−1^, and 53 RIU^−1^ were calculated for L/P = 0.33 in the Au-G hybrid sensors with prism (Figure 7a), cylindrical (Figure 7b), and cubic (Figure 7c) NPs, respectively. By comparing the sensitivity of Au-G hybrid sensors with different NPs shapes it was found that the L/P = 0.33 (L = 100 nm) offers larger sensitivity. It is known that the sensitivity results directly affect the FOM of the sensor [8]. The FOM based on the intensity shift at different L/P ratios and three different hybrid sensors are compared in Figure 8. It was found that the FOM was decreased on increasing the L/P from 0.17 to 1. As can be seen from this figure, for a fix L/P ratio the FOM was increased on increasing the refractive index from 1.333 to 1.341. The maximum FOM as large as 240 was achieved at L/P = 0.17 (NP size = 50 nm) (Figure 8c) in a hybrid sensor with cubic NPs. However, using prism and cylindrical NPs resulted in a FOM of 149 and 163 respectively at L/P = 0.17, as it is evident from Figure 8a,b.

The oscillations in the recorded sensitivity (Figure 7) and FOM (Figure 8) originated from the effect of the different environment permittivity (refractive index) on the magnitude of the recorded electric field at each resonance mode and the physical nature of the resonance mode and, hence, recording different intensities. For example, on using cylindrical NPs with 100 nm diameter, the intensity shift was 0.034 as the refractive index change from 1.333 to 1.334, while when the refractive index changes from 1.334 to 1.335 the intensity shift was 0.008, which results in a reduction in sensitivity and shows a reduction Figure 7b and Figure 8b.

Furthermore, at some L/Ps, a resonance wavelength shift up to 4.4 nm was also recorded in the hybrid sensors by using different NPs shapes at different L/P ratios and refractive index changes, as summarized in Table 1. From this table, it is clear that the maximum FOM of 390 was obtained in an Au-G hybrid sensor with cylinder NPs at L/P = 0.33 by increasing the refractive index from 1.334 to 1.335, whereas the maximum sensitivity as large as 4380 nm/RIU was obtained in a hybrid sensor with prism NPs at L/P = 0.33, which resulted in a FOM of 273 as the refractive index increased from 1.336 to 1.337. Detection at this level of refractive index change is capable of detecting a concentration at ng/mole and lower levels, as reported [28].

## 4. Conclusions 

Extinction properties, sensitivity, and FOM of the hybrid Au-G plasmonic sensors with different NPs shapes were systematically studied. The optical spectroscopy of the proposed sensors shows that the resonances phenomena were observed at near infrared region due to presence of graphene layers. It was shown that the resonance peaks were blue shifted by increasing the L/P ratio. Intensity sensitivity and FOM of all hybrid sensors were also studied for Δn = 0.001. A significant improvement in the FOM and sensitivity of the hybrid sensors was achieved. The maximum FOM as large as 240 and a high sensitivity as large as 55 RIU^−1^ were recorded that enhance the capability of the LSPR sensors in sensing and detection up to ng/mL level. The current results increase the capability of the LSPR sensors to compete the PSPR sensor. Further optimization is undertaken to enhance the ability of proposed hybrid structure to detect a liquid concentration at pg/mL and ag/m level.

## Figures and Tables

**Table 1:** Maximum sensitivity and FOM based on intensity and wavelength shift in different Au-G hybrid structures with prism, cylinder, and cubic NPs at specific changes in the refractive index.

NP	λ_resoance_ (nm)	Shift (nm)	n_1_	n_2_	S_λ_ (nm/RIU)	FOM
Prism	1805	4.38	1.336	1.337	4380	273
Cylindrical	1789	4.02	1.334	1.335	4020	390
Cubic	1765	4.34	1.334	1.335	4340	307
